# Prevalence of Antimicrobial Resistance (AMR) *Salmonella* spp. and *Escherichia coli* Isolated from Broilers in the East Coast of Peninsular Malaysia

**DOI:** 10.3390/antibiotics10050579

**Published:** 2021-05-13

**Authors:** Shamsaldeen Ibrahim, Loh Wei Hoong, Yip Lai Siong, Zaharuddin Mustapha, C. W. Salma C. W. Zalati, Erkihun Aklilu, Maizan Mohamad, Nor Fadhilah Kamaruzzaman

**Affiliations:** 1 Faculty of Veterinary Medicine, University Malaysia Kelantan, Pengkalan Chepa 16100, Kelantan, Malaysia; shams88ns@gmail.com (S.I.); salma.z@umk.edu.my (C.W.S.C.W.Z.); erkihun@umk.edu.my (E.A.); maizan.m@umk.edu.my (M.M.); 2 Faculty of Veterinary Science, University of Nyala, PO Box 155, Nyala 63311, South Darfur State, Sudan; 3 Rhone Ma Malaysia Sdn. Bhd., Lot 18A & 18B, Jalan 241, Seksyen 51A, Petaling Jaya 46100, Selangor, Malaysia; weihoong.loh@rhonema.com (L.W.H.); karen.yip@rhonema.com (Y.L.S.); 4 Department of Veterinary Services Pahang, Jalan Sri Kemunting 2, Kuantan 25100, Pahang, Malaysia; zaharuddin@dvs.gov.my

**Keywords:** broiler, antimicrobial resistance, *Salmonella species*, *E. coli*, Malaysia

## Abstract

*Salmonella species* (spp.) and *Escherichia coli* (*E. coli*) are the most common infectious pathogens in poultry. Antimicrobials are given either as growth promoters or as treatment, thereby increasing the possibility of the emergence of antimicrobial resistance (AMR). We determined the prevalence of AMR for both pathogens isolated from broiler farms in the East Coast of Peninsular Malaysia from 2018–2019. A total of 384 cloacal swabs were collected, followed by bacterial isolation, confirmation, and antimicrobial susceptibility tests. The overall prevalence of *Salmonella* spp. and *E. coli* were 6.5% and 51.8%, respectively. *Salmonella* spp. and *E. coli* displayed resistance towards the following antimicrobials: erythromycin (100% for both pathogens), chloramphenicol (76.2% and 84.5%, respectively), tetracycline (62% and 94.6%, respectively), ampicillin (47.7% and 87%, respectively), sulfamethoxazole/trimethoprim (42.9% and 83.3%, respectively), ciprofloxacin (4.8% and 23.8%, respectively), nalidixic acid (9.6% and 60.7%, respectively), streptomycin (19% and 66%, respectively), kanamycin (28.6% and 57%, respectively), cephalothin (0% and 11%, respectively), and gentamicin (0% and 20.2%, respectively). Multidrug resistance (MDR) was recorded in 82% of *Salmonella* spp. and 100% of *E. coli.* These findings demonstrate the high prevalence of AMR in both pathogens in broiler farms on the East Coast of Peninsular Malaysia. These findings could be attributed to the excessive use of antimicrobial agents by poultry farm owners. Enhanced control measures and a strong monitoring system should be urgently implemented in order to reduce the emergence of antimicrobial resistance.

## 1. Introduction

The poultry industry is the primary agricultural sector in Malaysia, contributing to 62.9% of the total gross domestic product (GDP) for the animal farming industry [1]. It has transformed with an increase in the number of products and an increasing number of integrators. Malaysians consume approximately 1.8 million chickens and 2.8 million eggs daily, which translates to an annual consumption of 31 kg of meat and 16.6 kg of eggs per capita. This is considered to be the highest meat consumed because of the large Muslim population and the higher price of other protein sources such as beef and mutton [2]. In Peninsular Malaysia, there are approximately 3200 broiler farms, including contract and independent farmers and large vertically integrated farms. Although the industry is expanding, the sector still faces many challenges, including infectious disease outbreak of avian salmonellosis and colibacillosis [3,4].

Avian salmonellosis is caused by the invasion and intestinal colonisation of *Salmonella* serovars, resulting in enteritis, septicemia, and animal mortality. *Salmonella* serovars, particularly *Salmonella* Enteritidis and *Salmonella* Typhimurium, can persist in chickens’ digestive tracts [5]. *Salmonellosis* is caused by non-typhoidal *Salmonella* and is typically characterised by gastroenteritis syndrome in humans [6]. *Salmonellosis* is the most frequent food-borne illness associated with consuming contaminated animals products, leading to health problems in humans and catastrophic economic impacts on the poultry industry [7]. Until now, more than 2600 serovars of *Salmonellae* have been identified worldwide, and most of them causing illness in humans and animals [8]. According to the World Health Organization (WHO), non-typhoidal *Salmonella* is estimated to cause more than 90 million illnesses worldwide, and accounts for approximately 155,000 deaths each year. The Centers for Disease Control and Prevention (CDC) has reported more than 40,000 *Salmonellosis* infections in the United States annually [9]. In parallel, the European Centre for Disease Prevention and Control (ECDC) has reported *Salmonellosis* as the second leading cause of gastrointestinal infection, with a confirmed case rate of 20.4 cases per 100,000 individuals [10]. China alone estimated that 22.2% of their food-borne illness are related to *Salmonellosis* [10].

In parallel, avian colibacillosis is another significant infectious disease caused by pathogenic *E. coli* strains, and causes massive economic losses to the poultry industry because of the high morbidity, mortality, and increasing cost for treatment and prevention [11]. The condition is characterized by respiratory infection, yolk sac infection, coli granuloma, enteritis, cellulitis omphalitis, swollen head syndrome, septicemia, polyserositis, and salpingitis [12]. The severity of the disease depends on the host’s health status, predisposing factors, and virulence of the *E. coli* strain. In recent report estimated that 30% of broiler flocks In the US are affected by subclinical colibacillosis [13]. *E. coli* normally found In gastrointestinal of broiler chickens, mucosal surfaces, and poultry environment [14]. Pathogenic *E. coli* strains such as O157 and O104 can cause significant human health problems [15]. The *E. coli* O104:H4 outbreak alone caused a loss of US$1.3 billion for Germany’s farmers and industries, and required payments of US$236 million in emergency aid to 22 European Union member states in 2011 [15]. Several strategies have been implemented to control colibacillosis, which include the administration of vaccines. Koutsianos et al. recently assessed on the impact of different vaccination program regarding bacillosis protection in conventional pullets. The authors discovered that animals that were immunized twice with a three-valance autogenous vaccine—first with the commercial vaccine, followed by the autogenous vaccine—were significantly protected by bacillosis [16].

Other than vaccines, antimicrobials are used widely for the treatment and prevention of infectious disease in livestock. These practices, in part, are associated with increased rates of antimicrobial resistance among pathogens isolated from animals. There is growing concern that widespread antimicrobial use has led to the emergence of resistant organisms to most or all antimicrobials [17]. Antimicrobial resistance bacteria isolated from production animals may lead to therapy failure and economic losses to farmers. The transmission of resistant bacteria to humans can potentially lead to treatment failure and death [18]. A recent report projected that approximately 50 million human deaths in 2050 would be due to antimicrobial resistance [18]. Antimicrobial resistance is a big challenge for Malaysian public health. Increasing cases of treatment failure in humans and animals have been reported in recent years, showing that the pathogens do not respond to the antimicrobials administered for the treatment [19].

We found minimal data on the prevalence of antimicrobial resistance (AMR) in *Salmonella* spp. and *E. coli* in the poultry industry in the East Coast of Peninsular Malaysia. Thus, the present study aimed to determine the prevalence of AMR in *Salmonella* spp. and *E. coli* isolated from broiler farms in three states—Kelantan, Terengganu, and Pahang—located in the East Coast of Peninsular Malaysia.

## 2. Results

### 2.1. Prevalence of Salmonella spp. and E. coli in Broiler Farms in Kelantan, Terengganu, and Pahang

Of the 384 samples, a total of 25 *Salmonella* spp. and 199 *E. coli* were isolated, with an overall prevalence of 6.6% and 51.8%, respectively. In Kelantan, Terengganu, and Pahang, the prevalence of *Salmonella* spp. was 7%, 6.5%, and 5.8%, respectively, while the prevalence of *E. coli* was 50%, 48.3%, and 58%, respectively. Table 1 summarizes the results for the prevalence and distribution of *Salmonella* spp. and *E. coli* isolated from broilers in the three states.

### 2.2. Salmonella and E. coli Susceptibility towards Antimicrobial Tested

#### 2.2.1. Overall *Salmonella* and *E. coli* Susceptibility towards Antimicrobial Tested

To determine *Salmonella* spp. and *E. coli* isolates’ susceptibility towards the selected antimicrobials, an antimicrobial susceptibility test was performed using disc diffusion methods. *Salmonella* spp. and *E. coli* displayed resistance towards the following antimicrobials: erythromycin (100% for both pathogens), chloramphenicol (76.2% and 84.5%, respectively), tetracycline (62% and 94.6%, respectively), ampicillin (47.7% and 87%, respectively), sulfamethoxazole/trimethoprim (42.9% and 83.3%, respectively), ciprofloxacin (4.8% and 23.8%, respectively), nalidixic acid (9.6% and 60.7%, respectively), streptomycin (19% and 66%, respectively), kanamycin (28.6% and 57%, respectively), cephalothin (0% and 11%, respectively), and gentamicin (0% and 20.2%, respectively). All *Salmonella* and *E. coli* isolates were sensitive to the colistin antimicrobial. Table 2 summarizes *Salmonella* and *E. coli* susceptibility towards all of the antimicrobials tested.

#### 2.2.2. Distribution of Antimicrobial Resistance in Kelantan, Terengganu, and Pahang

**Figure 1:**
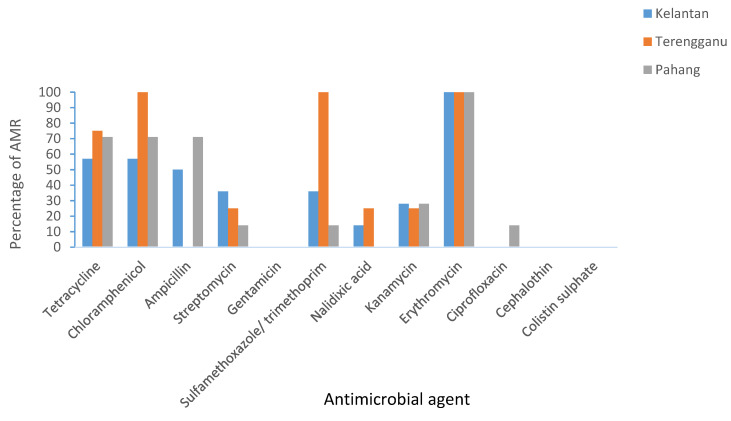
Prevalence of AMR in *Salmonella* spp. isolated from broilers in Kelantan, Terengganu, and Pahang.

**Figure 2:**
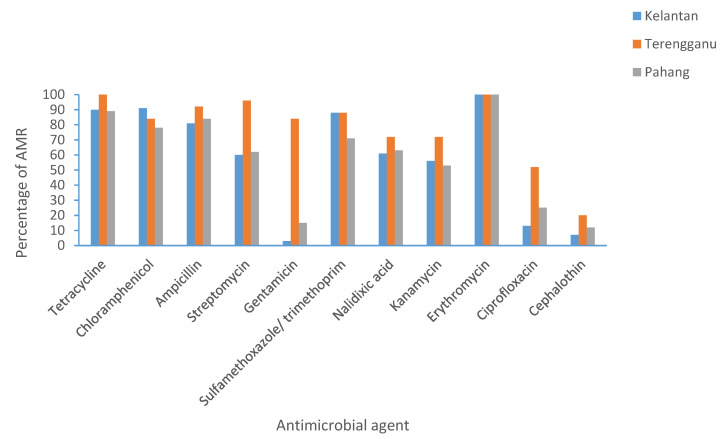
Prevalence of antimicrobial resistance (AMR) in *E. coli* isolated from broilers in Kelantan, Terengganu, and Pahang.

In Kelantan, >50% *Salmonella* spp. was found to have resistance towards tetracycline, chloramphenicol, ampicillin, and erythromycin. While >50% *E. coli* was found to have resistance towards all of the antimicrobials tested, except for ciprofloxacin, cephalothin, and colistin sulphate. In Terengganu, >50% *Salmonella* spp. was found to have resistance towards tetracycline, chloramphenicol, sulfamethoxazole/trimethoprim, and erythromycin. While >50% *E. coli* isolates demonstrated resistance towards almost all of the antimicrobials, except cephalothin and colistin sulphate. Finally, in Pahang, >50% *Salmonella* spp. was found to have resistance towards tetracycline, chloramphenicol, ampicillin, and erythromycin. While >50% *E. coli* isolates demonstrated resistance towards almost all antimicrobials, except cephalothin and colistin. In summary, the highest resistance for *Salmonella* and *E. coli* for all three states was towards tetracycline, chloramphenicol, and erythromycin. Figure 1 and Figure 2 summarize the percentage of AMR in Kelantan, Terengganu, and Pahang.

#### 2.2.3. *Salmonella* spp. and *E. coli* MDR Profile and Multiple Antimicrobial Resistance (MAR) Index

The MDR profiles for *Salmonella* spp. and *E. coli* were also tabulated. A total of 81% of *Salmonella* spp. isolates showed a multidrug resistance profile (resistance to >1 antimicrobial). This profile included 4.8% isolates resistant to six to eight antimicrobials, 14.2% to five antimicrobials, 42.8% to four antimicrobials, and 9.5% to three antimicrobials. The most predominant MDR profile for *Salmonella* spp. was TE-C-AMP-E, TE-C-SXT-E, C-AMP-K-E, and C-AMP-SXT-E.

In parallel, 5.9% of *E. coli* were resistant to ten antimicrobials, 10.7% to nine antimicrobials, 21.4% to eight antimicrobials, 20.2% to seven antimicrobials, 17.2% to six antimicrobials, 12.5% to five antimicrobials, 6.5% to four antimicrobials, and 3.5% to three antimicrobials. The MDR profile *E. coli* isolates showed varieties of the AMR profile, where 56 different MDR profiles were recorded. The most predominant antibiotypes were TE-C-AMP-S-SXT-NA-K-E, TE-C-AMP-S-SXT-NA-E, and TE-C-SXT-NA-E-CIP. Table 3 summarizes the MDR profiles for *Salmonella* spp. and *E. coli*.

MAR index helped analyze health risk and check the extent of antimicrobial resistance. The MAR index was calculated for both of the *Salmonella* and *E. coli* isolates. The analysis showed that 71% of *Salmonella* isolates have MAR > 0.2. In comparison, 96% *E. coli* isolates showed an MAR index > 0.2 (Table 3), suggesting that the isolates originated from a high-risk source of contamination where antimicrobials are commonly used.

## 3. Discussion

Increasing AMR cases in humans, in part, has been correlated with the transmission of pathogens from animals to humans. Here, we found that *Salmonella* and *E. coli* isolated from broilers in the East Coast of Peninsular Malaysia displayed multidrug resistance towards commonly used antimicrobials used in animals and humans. We also found that the majority of isolated *Salmonella* and *E. coli* had an MAR index > 0.2.

*Salmonella* spp. and *E. coli* are the predominant bacteria associated with bacterial infection in poultry. These organisms are known to result in severe poultry health problems, leading to mortality, reduced production, and increased expense in preventing and treating the disease. A broad diversity of antimicrobials is used to raise poultry in most countries, mainly through the oral route, to prevent and treat disease and enhance growth and productivity [20]. Our study’s findings agree with a study conducted in Selangor, Malaysia, that reported a high prevalence of *E. coli* (60%) compared with only 7.5% of *Salmonella* spp. isolated from the same samples [21]. Another study reported that the presence of *Salmonella* isolated from backyard chickens in Malaysia was 2.5% [22]. A low prevalence of *Salmonella* isolated from poultry was also reported in other countries such as Nigeria (2%) and European countries [23]. Interestingly, the same trend does not appear to be so in Bangladesh, as a study showed a high prevalence (48%) of *Salmonella* isolated from poultry [24].

Antimicrobial resistance in chickens is a common problem in Malaysia and other developing countries as a result of antimicrobials used as feed additives and the prophylactic treatment of infectious diseases. Our study found 100% *Salmonella* and *E. coli* resistance towards erythromycin. This finding agrees with another study conducted in Bangladesh, which reported the same resistance trend [24]. Our study also found a high prevalence of multidrug-resistant *Salmonella* and *E. coli* isolates, which is in agreement with previous studies conducted in Malaysia [21]. These findings provide evidence for the emergence for the antimicrobial resistance of *Salmonella* spp. and *E. coli* in poultry farms in Malaysia. It is interesting to note that all *Salmonella* and *E. coli* isolates were susceptible to colistin. However, a recent study conducted in the same region detected the *MCR-1* gene, which was found to encode colistin-resistant in *E. coli* isolated in raw chicken meat in Kelantan, Malaysia [25]. It is important to note that the study conducted by Aklilu and Raman used a molecular biology method that is known to be more sensitive than the disc diffusion method.

**Figure 3:**
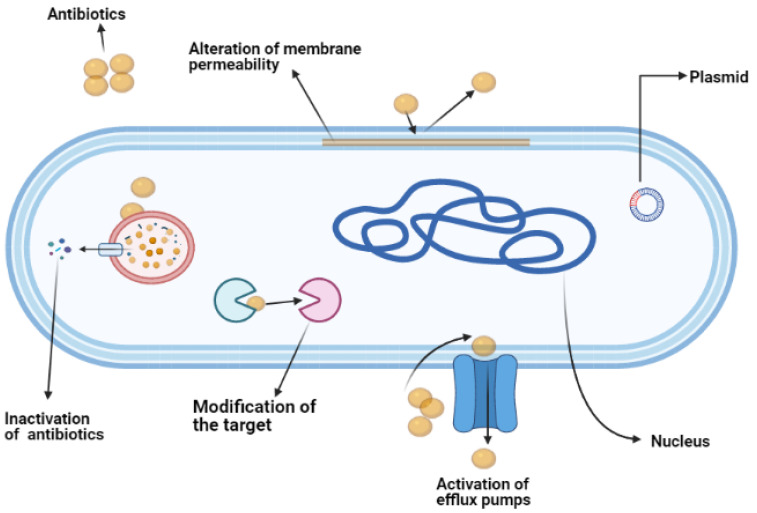
The main mechanisms of antibiotics/antimicrobial resistance in bacteria.

In general, antimicrobial resistance in bacteria occurs when the bacteria develop a mechanism to survive in the presence of antimicrobials [26]. This survival is attributed to the ability of the bacteria to limit the penetration of antimicrobials, modify the drug or the drug target, and express an efflux system to reduce the drug concentration within the cells [27]. This resistance develops either because of intrinsic or acquired mechanisms [28]. An intrinsic mechanism is a condition where the bacteria is naturally resistant towards the antimicrobial. A good example of an intrinsic mechanism is the physical structure of the outer membrane of Gram-negative bacteria, which limits the penetration of vancomycin, which is effective against Gram-positive bacteria. Intrinsic resistance also includes the expression of general porins that efflux the antimicrobials from the bacterial cells. Genes that express the innate resistance characteristic are usually encoded in the genome of the bacteria [28]. In contrast, bacteria can also develop antimicrobial resistance via acquired mechanisms. The acquired resistance occurs through the transfer of mobile resistance genes (for example, plasmid) from other resistance bacteria. Additionally, mutations in the gene could also cause changes in the drug target when the bacteria is constantly under pressure after being exposed to the antimicrobial [28]. The main mechanism of antimicrobial resistance is summarized in Figure 3.

In conclusion, this finding indicates the high prevalence of multi-drugs resistant *Salmonella* spp. and *E. coli* in poultry farms in the East Coast of Peninsular Malaysia. This finding, in part, could be attributed to the excessive use of antimicrobial agents by poultry farm owners, which are potentially harmful to public health. Control measures and a strong monitoring system should be urgently advocated for and implemented in Malaysia in order to reduce AMR emergence. In addition, further research on alternatives to antimicrobials, good animal husbandry practices, and biosecurity should be encouraged in order to replace the application of existing antimicrobials in animal health.

## 4. Materials and Methods

### 4.1. Ethical Statement

The current study was conducted at the Zoonotic Laboratory, Faculty of Veterinary Medicine, Universiti Malaysia Kelantan. The study protocols, procedures, and consent form were approved by the Institutional Animal Care and Use Committee of Universiti Malaysia Kelantan (UMK/FPV/ACUE/PG/2/2019).

### 4.2. Sampling Method and Sample Size Determination

**Figure 4:**
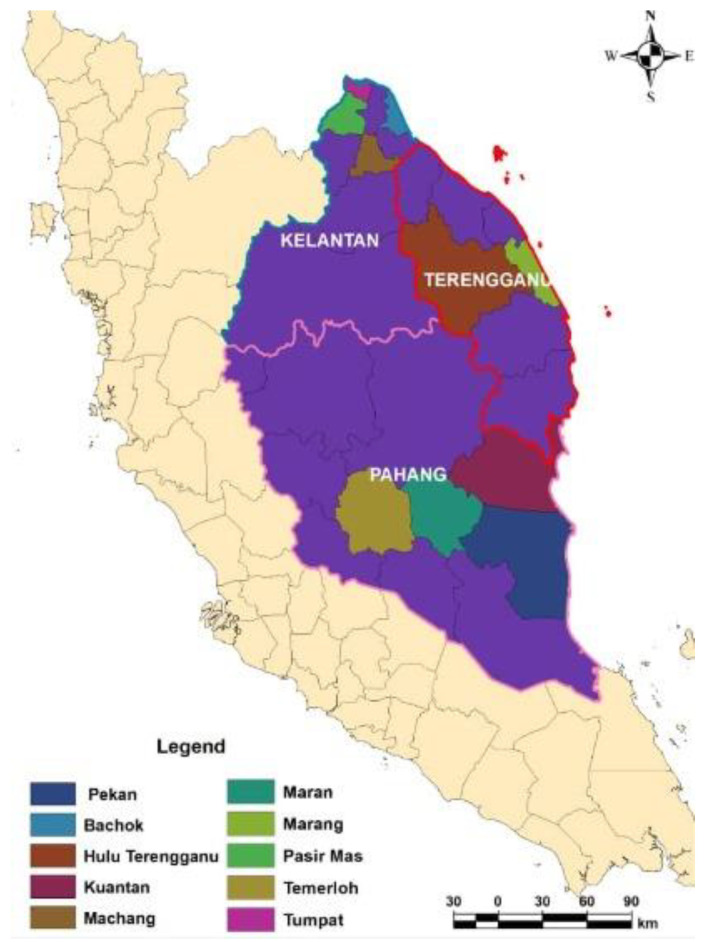
Location of the study area. The samples were collected from 10 districts in Kelantan, Terengganu, and Pahang, off the East Coast of Peninsular Malaysia. The map was created using ArcGIS v. 7 (Esri Inc., Redlands, CA, USA).

Cloacal swabs were obtained from broilers from 30 different farms in Kelantan, Terengganu, and Pahang (Figure 4), located in the East Coast of Peninsular Malaysia. The farms were selected based on the list of broiler farms provided by the Department of Veterinary Services, Malaysia. Farm selection was performed by the multistage random selection method. Cloacal swabs were collected aseptically using sterile swabs with Ames transport media. Following sample collection, the samples were immediately transported back to the laboratory in cold storage for further processing. The sample size was determined by using StatCalc from Epi-Info (7) using a formula based on Thrusfield (2007) [29].

### 4.3. Bacteria Isolation and Identification

Before bacteria isolation, pre-enrichment was performed by inoculating the swabs into buffered peptone water (BPW; Oxoid, Basingstoke, UK), followed by incubation at 37 °C for 24 h. For *E. coli* isolation, the enriched BPW was streaked on MacConkey agar plates (MAC; Oxoid, UK) and incubated aerobically for 24 h at 37 °C. Suspected lactose fermentative *E. coli* colonies were sub-cultured on eosin methylene blue agar (EMB; Oxoid, UK) for another 24 h at 37 °C. The suspected *E. coli*, which displayed green metallic shine colonies, were further subjected to biochemical testing. Colonies that exhibited acid slant, acid butt, and no hydrogen sulphate production on triple sugar iron, indole, and decarboxylase positive, regardless of motility, were considered to be *E. coli*, and were subcultured and stored in glycerol stock and kept at −80 °C until ready for further use [30].

For the *Salmonella* isolation, 0.1 mL of BPW mixture were inoculated in Rappaport-Vassiliadis Soya Pepton Broth (RVS; Oxoid, UK) at 42 °C for 24 h for selective enrichment. Following that, the RVS mixture was streaked in xylose-lysine-deoxycholate agar (XLD; Oxoid, UK) and incubated aerobically for 24 h at 37 °C. After 24 h, the plates was examined for the presence of suspected *Salmonella* spp. The suspected colonies were subjected to a biochemical test followed by the latex agglutination test using the commercially available polyvalent antisera (Oxoid *Salmonella* test kit DR1108A) to screen for the *Salmonella* flagellar antigen. Briefly, a loop full of suspected colonies were emulsified with one drop of 0.85% sodium chloride on the reaction card to produce the smooth suspension. A drop of *Salmonella* latex reagent was added and mixed with the organism suspension with the clean mixing stick. The *Salmonella* isolates caused agglutination in the reaction [10].

### 4.4. Antimicrobial Sensitivity Test

The antimicrobial sensitivity for all of the isolates was determined through the standard antimicrobial disk diffusion test protocol by the Clinical and Laboratory Standard Institute (CLSI) [31]. The following antimicrobial commercial discs from Oxoid, UK were used in this study: tetracycline(TE; 30 μg), chloramphenicol (C; 30 μg), ampicillin (AMP; 10 μg), cephalothin (CL; 30 μg), streptomycin (S; 10 μg), gentamicin (CN; 10 μg), sulfamethoxazole/trimethoprim (SXT; 25 μg), nalidixic acid (NA; 30 μg), ciprofloxacin (5 μg), erythromycin(E; 15 μg), kanamycin (K; 30 μg), and clostine sulphate (CT; 10 µg). All of the selected antimicrobials are commonly used for the treatment of infections associated with *E. coli* and *Salmonella* based on the recommendation by World Organization for Animal Health. Briefly, 0.5 McFarland bacterial suspension was prepared and plated on the agar surface. Six paper discs were placed onto each agar plate using a dispenser. The plate was incubated at 37 °C for 18 h. The resulting zones of inhibition (ZOI) were measured in millimeters using a vernier caliper and the measurements were rounded off to the nearest whole number. The antimicrobial sensitivity profiles of the isolates were determined following the zone of the inihibition diameter breakpoints and interpretative categories (susceptible, intermediate, or resistant) for Enterobacteriaceae, as recommended by CLSI [32].

### 4.5. Determination of Multiple Antimicrobial Resistance (MAR) Indexes

The MAR was calculated as reported by Christopher and Ali (2013) as follows [33]:

$$\mathrm{MAR}\;\mathrm{index}=\frac{\;\;\mathrm{Number}\;\mathrm{of}\;\mathrm{antimicrobials}\;\mathrm{to}\;\mathrm{which}\;\mathrm{the}\;\mathrm{isolate}\;\mathrm{showed}\;\mathrm{resistance}}{\mathrm{Number}\;\mathrm{of}\;\mathrm{total}\;\mathrm{antibiotics}\;\mathrm{exposed}\;\mathrm{to}\;\mathrm{the}\;\mathrm{isolate}}$$

The results were interpreted according to Nandi and Mandal: an MAR index ≤ 0.2 was considered to be low risk, while ≥0.2 indicated a high risk of antimicrobial contamination [33].

### 4.6. Statistical Analysis

The results were analyzed statistically using Graph Pad Prism version 8. The level of significance was determined at a 95% confidence level and at *p* < 0.05.

## Figures and Tables

**Table 1:** Prevalence and distribution of *Salmonella* spp. and *E. coli* isolated from broilers in Kelantan, Terengganu, and Pahang.

State/Locality	No. of Samples	Prevalence of *Salmonella* spp.	Prevalence of *E. coli*
Kelantan			
Machang	40	0%	50%
Bachok	40	15%	45%
Tumpat	40	12.5%	62.5%
Pasir Mas	40	7.5%	37.5%
Jeli	40	0%	55%
Total	200	**7%**	**50%**
**Terengganu**			
Marang	30	0%	33.3%
Hulu Terengganu	30	13.3%	63.3%
Total	60	6.5%	48.3%
**Pahang**			
Kuantan	32	0%	65.6%
Pekan	32	0%	59.4%
Maran	30	13.3%	33.3%
Temerloh	30	10%	66.6%
Total	120	5.8%	58.0%
Overall	384	6.5%	51.8%

**Table 2:** *Salmonella* spp. and *E. coli* susceptibility towards all of the antimicrobials tested.

Pathogens/Antimicrobials	Susceptible (%)		Intermediate (%)		Resistant (%)	
	*Salmonella*	*E. coli*	*Salmonella*	*E. coli*	*Salmonella*	*E. coli*
Tetracycline	38	5.3	0	0	62	94.6
Chloramphenicol	23.8	14.8	0	0.5	76.2	84.5
Ampicillin	52.3	12	0	0.5	47.7	87.5
Streptomycin	76.1	31	4.7	3	19	66
Gentamicin	100	75.6	0	4.2	0	20.2
Sulfamethoxazole/trimethoprim	57.1	16	0	0.5	42.9	83.3
Nalidixic acid	90.4	39.3	0	0	9.6	60.7
Kanamycin	71.4	43	0	0	28.6	57
Erythromycin	0	0	0	0	100	100
Ciprofloxacin	95.2	72	0	4.2	4.8	23.8
Cephalothin	100	87	0	2	0	11
Colistin sulphate	100	100	0	0	0	0

**Table 3:** Antimicrobial resistance patterns and multiple resistance index (MAR) in *Salmonella* spp. and *E. coli* isolates.

No of Antimicrobials	*Salmonella*			*E. coli*		
	MDR Profile	% of Isolates	MAR Index	MDR Profile	% of Isolates	MAR Index
12	N/A	N/A	N/A	N/A	N/A	N/A
11	N/A	N/A	N/A	TE-C-AMP-S-CN-SXT-NA-K-E-CIP-CL	1.2	0.9
10	N/A	N/A	N/A	TE-C-AMP-S-CN-SXT-NA-K-E-CIP, TE-C-S-CN-SXT-NA-K-E-CIP-CL, TE-C-AMP-S-CN-NA-K-E-CIP-CL	5.9	0.8
9	N/A	N/A	N/A	TE-C-AMP-S-CN-SXT-NA-K-E TE-C-AMP-S-SXT-NA-K-E-CIP TE-C-AMP-S-SXT-K-E-CIP-CL TE-C-AMP-S-NA-K-E-CIP-CL C-AMP-S-CN-SXT-NA-K-E-CIP TE-C-AMP-S-CN-SXT-NA-E-CIP	10.7	0.7
8	TE-C-AMP-S-SXT-K-E-CIP	4.8	0.7	TE-C-AMP-S-CN-SXT-E-CL TE-C-AMP-S-SXT-NA-K-E TE-C-AMP-S-SXT-NA-K-E TE-C-AMP-S-CN-SXT-K-E TE-C-AMP-S-CN-SXT-K-E TE-C-AMP-S-NA-K-E-CIP TE-C-AMP-S-CN-NA-K-E TE-AMP-S-CN-SXT-NA-K-E	21.4	0.6
7	TE-C-S-SXT-NA-K-E	4.8	0.6	TE-C-AMP-S-SXT-NA-E TE-C-AMP-SXT-NA-K-E TE-AMP-S-SXT-NA-K-E TE–C-S-SXT-NA-K-E TE-AMP-S-SXT-NA-K-E TE-C-AMP-CN-SXT-K-E TE-C-AMP-S-SXT-K-E TE-C-SXT-NA-K-E-CIP TE-C-AMP-SXT-NA-E-CIP TE-C-AMP-NA-E-CIP-CL	20.2	0.5
6	TE-C-AMP-S-SXT-E	4.8	0.5	TE-C-AMP-SXT-K-E TE-C-AMP-S-SXT-E TE-C-SXT-NA-E-CIP TE-C-AMP-SXT-NA-E TE-AMP-SXT-NA-K-E TE-C-AMP-NA-E-CIP TE-S-CN-SXT-K-E	17.2	0.5
5	S-SXT-NA-K-E TE-C-AMP-K-E	14.2	0.4	TE-C-AMP-SXT-E TE–C-S-SXT-E TE-C-AMP-K-E AMP-NA-E-CIP-CL TE-AMP-SXT-NA-E TE-AMP-SXT-K-E TE-AMP-S-SXT-E TE-AMP-NA-E-CIP C-AMP-SXT-K-E TE-AMP-S-NA-E	12.5	0.4
4	TE-C-AMP-E TE-C-SXT-E C-AMP-K-E C-AMP-SXT-E	42.8	0.3	TE-S-SXT-E TE-C-AM-E C-AMP-SXT-E TE-AMP-SXT-E TE-AMP-E-CL AMP-K-E-CL TE-C-SXT-E	6.5	0.3
3	SXT-C-E TE-C-E	9.5	0.2	TE-AMP-E S-NA-E TE-C-E	3.5	0.2
2	N/A	N/A	N/A	AMP-E	0.5	0.1
1	E	19	0.08	N/A	0	0

TE—tetracycline; C—chloramphenicol; AMP—ampicillin; CL—cephalothin; S—streptomycin; CN—gentamicin; SXT—sulfamethoxazole/trimethoprim; NA—nalidixic acid; CIP—ciprofloxacin; E—erythromycin; K—kanamycin.

## Data Availability

The data used to support the findings of this study are included in this study.

## References

[1] Amna N., Mohammad A., Rosali M.H. *The Development and Future Direction of Malaysia’s Livestock Industry*. FFTC-Agricultural Policy Platform (FFTC-AP) 2020. https://ap.fftc.org.tw/article/960.

[2] Orissa International (2017). Poultry Sector in South East Asia Iowa Economic Development Authority.

[3] Abu Daud N.H.B., Htin N.N., Abba Y., Paan F.H., Kyaw T., Khaing A.T., Jesse F.F.A., Mohammed K., Adamu L., Tijjani A. (2014). An Outbreak of Colibacillosis in a Broiler Farm. J. Vet. Adv..

[4] Chuah L.O., Shamila Syuhada A.K., Mohamad Suhaimi I., Farah Hanim T., Rusul G. (2018). Data on antibiogram and resistance genes harboured by *Salmonella* strains and their Pulsed-field gel electrophoresis clusters. Data Br..

[5] Huang T.-M., Lin T.L., Wu C.C. (2009). Antimicrobial Susceptibility and Resistance of Chicken *Escherichia coli*, *Salmonella* spp., and *Pasteurella multocida* Isolates. Avian Dis..

[6] Antunes P., Mourão J., Campos J., Peixe L. (2016). Salmonellosis: The role of poultry meat. Clin. Microbiol. Infect..

[7] Mouttotou N., Ahmad S., Kamran Z., Koutoulis K.V. (2016). Prevalence, Risks and Antibiotic Resistance of *Salmonella* in in Poultry Poultry Production *Salmonella* Production Chain. Current Topics in Salmonella and Salmonellosis.

[8] Nero L.A. (2018). Highly Prevalent Multidrug-Resistant *Salmonella* from Chicken and Pork Meat at Retail Markets. Front. Microbiol..

[9] Bula-Rudas F.J. (2015). *Salmonella* Infections in Childhood. Adv. Pediatr..

[10] Kuang X., Hao H., Dai M., Wang Y., Ahmed J., Liu Z., Zonghui Y. (2015). Serotypes and antimicrobial susceptibility of *Salmonella* spp. isolated from farm animals in China. Front. Microbiol..

[11] Bin Kim Y., Young Yoon M., Su Ha J., Won Seo K., Bi Noh E., Son S.H., Lee Y.J. (2020). Molecular characterisation of avian pathogenic Escherichia coli from broiler chickens with colibacillosis. Poult. Sci..

[12] Kabir S.M.L. (2010). Avian Colibacillosis and Salmonellosis: A Closer Look at Epidemiology, Pathogenesis, Diagnosis, Control and Public Health Concerns. Int. J. Environ. Res. Public Health.

[13] Fancher C.A., Zhang L., Kiess A.S., Adhikari P.A., Dinh T.T.N., Sukumaran A.T. (2020). Avian pathogenic Escherichia coli and clostridium perfringens: Challenges in no antibiotics ever broiler production and potential solutions. Microorganisms.

[14] Ricke S.C., Rothrock M.J. (2020). Gastrointestinal microbiomes of broilers and layer hens in alternative production systems. Poult. Sci..

[15] Yang S.C., Lin C.H., Aljuffali I.A., Fang J.Y. (2017). Current pathogenic Escherichia coli food-borne outbreak cases and therapy development. Arch. Microbiol..

[16] Koutsianos D., Gantelet H., Franzo G., Lecoupeur M., Thibault E., Cecchinato M., Koutoulis K.C. (2020). An assessment of the level of protection against colibacillosis conferred by several autogenous and/or commercial vaccination programs in conventional pullets upon experimental challenge. Vet. Sci..

[17] Teillant A., Laxminarayan R. (2015). Economics of Antibiotic Use in USA. Swine and Poultry Production. Choices.

[18] Van den Bogaard E.A., Stobberingh E.E. (1999). Antibiotic Usage in Animals. Drugs.

[19] O’Neill J. (2016). Tackling Drug-Resistant Infections Globally: Final Report and Recommendations. Review on Antimicrobial Resistance.

[20] Alreshidi M.N., Neela M.A., Alsharari V., Hamat A.S., Alsalamah R.A., Atshan A.A., Alzoghaibi S.S., Shamsudin O. (2017). Molecular typing and antibiotic resistance patterns of methicillin-resistant Staphylococcus aureus isolates from clinical samples in Malaysia: An update. Trop. Biomed..

[21] Nhung N.T., Chansiripornchai N., Carrique-Mas J.J. (2017). Antimicrobial Resistance in Bacterial Poultry Pathogens: A Review. Front. Vet. Sci..

[22] Geidam Y., Zakaria Z., Aziz S.A., Bejo S.K., Abu J., Omar S. (2012). High Prevalence of Multi-drug Resistant Bacteria in Selected Poultry Farms in Selangor, Malaysia. Asian J. Anim. Vet. Adv..

[23] Jajere S.M., Hassan L., Aziz S.A., Zakaria Z., Abu J., Nordin F., Faiz N.M. (2015). *Salmonella* in native ‘village’ chickens Gallus domesticus Prevalence and risk factors from farms in South-Central Peninsular Malaysi. Poult. Sci..

[24] Gonçalves-Tenório A., Nunes Silva B., Rodrigues V., Cadavez V., Gonzales-Barron U. (2018). Prevalence of pathogens in poultry meat: A meta-analysis of European published surveys. Foods.

[25] Islam J., Ahmed T., Hasan K. (2017). Isolation and identification of *Salmonella* spp. from broiler and their antibiogram study in Sylhet, Bangladesh. J. Appl. Biol. Biotechnol..

[26] McManus M.C. (1997). Mechanisms of bacterial resistance to antimicrobial agents. Am. J. Health Syst. Pharm..

[27] Reygaert W.C. (2018). An overview of the antimicrobial resistance mechanisms of bacteria. AIMS Microbiol..

[28] Tenover F.C. (2006). Mechanisms of antimicrobial resistance in bacteria. Am. J. Infect. Control.

[29] Thrusfield M.V. (2007). Sample size determination. Veterinary Epidemiology.

[30] Ajijur M., Rahman M.M., Amin R., Begum I.A., Fries R., Husna A., Khairalla A.S., Badruzzaman A.T.M., Zowalaty M.E.E., Lampang K.N. (2019). Susceptibility and Multidrug Resistance Patterns of Escherichia coli Isolated from Cloacal Swabs of Live Broiler Chickens in Bangladesh. Pathogens.

[31] CLSI (2016). Clinical and Laboratory Standards Institute: Performance Standards for Antimicrobial Susceptibility Testing Supplement M100S.

[32] Christopher A.F., Hora S., Ali Z. (2013). Investigation of plasmid profile, antibiotic susceptibility pattern multiple antibiotic resistance index calculation of *Escherichia coli* isolates obtained from different human clinical specimens at tertiary care hospital in Bareilly-India. Ann. Trop. Med. Public Health.

[33] Akande E.B., Abodunrin T.F., Oladejo B.O., Oladunmoye M.K. (2019). Antibiogram and plasmid profiling of resistance bacteria isolated from the blood of Hepatitis C Virus positive individuals. J. Microbiol. Exp..

